# Diversity and Antimicrobial Activities of Actinobacteria Isolated from Tropical Mangrove Sediments in Malaysia

**DOI:** 10.1155/2014/698178

**Published:** 2014-08-05

**Authors:** Learn-Han Lee, Nurullhudda Zainal, Adzzie-Shazleen Azman, Shu-Kee Eng, Bey-Hing Goh, Wai-Fong Yin, Nurul-Syakima Ab Mutalib, Kok-Gan Chan

**Affiliations:** ^1^Jeffrey Cheah School of Medicine and Health Sciences, Monash University Malaysia, 46150 Bandar Sunway, Selangor Darul Ehsan, Malaysia; ^2^Division of Genetics and Molecular Biology, Institute of Biological Sciences, Faculty of Science, University of Malaya, 50603 Kuala Lumpur, Malaysia; ^3^School of Science, Monash University Malaysia, 46150 Bandar Sunway, Selangor Darul Ehsan, Malaysia; ^4^UKM Medical Molecular Biology Institute (UMBI), UKM Medical Centre, Bandar Tun Razak, 56000 Cheras, Kuala Lumpur, Malaysia

## Abstract

The aim of this study was to isolate and identify Actinobacteria from Malaysia mangrove forest and screen them for production of antimicrobial secondary metabolites. Eighty-seven isolates were isolated from soil samples collected at 4 different sites. This is the first report to describe the isolation of *Streptomyces, Mycobacterium, Leifsonia, Microbacterium, Sinomonas, Nocardia, Terrabacter, Streptacidiphilus, Micromonospora, Gordonia*, and *Nocardioides* from mangrove in east coast of Malaysia. Of 87 isolates, at least 5 isolates are considered as putative novel taxa. Nine *Streptomyces* sp. isolates were producing potent antimicrobial secondary metabolites, indicating that *Streptomyces* isolates are providing high quality metabolites for drug discovery purposes. The discovery of a novel species, *Streptomyces pluripotens* sp. nov. MUSC 135^T^ that produced potent secondary metabolites inhibiting the growth of MRSA, had provided promising metabolites for drug discovery research. The biosynthetic potential of 87 isolates was investigated by the detection of polyketide synthetase (PKS) and nonribosomal polyketide synthetase (NRPS) genes, the hallmarks of secondary metabolites production. Results showed that many isolates were positive for PKS-I (19.5%), PKS-II (42.5%), and NRPS (5.7%) genes, indicating that mangrove *Actinobacteria* have significant biosynthetic potential. Our results highlighted that mangrove environment represented a rich reservoir for isolation of *Actinobacteria*, which are potential sources for discovery of antimicrobial secondary metabolites.

## 1. Introduction

Actinobacteria represent a significant component of the microbial population in most soils including the mangrove region [1]. This phylum of bacteria has been extremely useful to the pharmaceutical industry due to their seemingly unlimited capacity to produce secondary metabolites with diverse biological activities and chemical structures [1–4]. Approximately 50% of Actinobacteria are from the genus* Streptomyces*, and approximately 75% of commercially useful antibiotics are derived from this genus [5].

In recent years, the chances of discovering novel biologically active molecules from various known soil bacteria (including Actinobacteria) have reduced, implying that a saturation effect could be occurring. The isolation of known Actinobacteria such as* Streptomyces* from various environments was found to be producing similar compounds [6]. Furthermore, the emergence of multidrug resistant pathogenic bacteria such as MRSA and fungus has resulted in critical demand for new natural products and chemical compounds in pharmacology, which in turn has made the exploration of poorly exploited areas such as the mangrove environments essential to discover novel Actinobacteria and novel metabolites [1, 7, 8].

The mangrove forests are highly productive ecosystems that provide vital protections to the coastline located in the tidal zones in the tropical and subtropical areas. These ecosystems are habitat to diverse flora and fauna of marine, freshwater, and terrestrial species [9]. In contrast to the well-documented species diversity of larger animals and plants in these mangrove ecosystems, the diversity of microbial community in the mangrove environments needs to be improved [1, 10, 11].

The constant changes in the environmental factors such as tidal gradient and salinity in the mangrove environments are understood to be the driving force for metabolic pathway adaptations that could lead to the production of unusual metabolites. Therefore, there had been increasing exploitation of the mangrove microorganism resources [1, 12]. Many researchers discovered that the poorly explored mangrove environments contain high populations of novel Actinobacteria, as demonstrated by the isolation of* Asanoa iriomotensis* [13],* Nonomuraea maheshkhaliensis* [14], and* Streptomyces xiamenensis* [10]. Furthermore, many strains are also prolific producers of useful antibiotics [1, 11, 15]. Many Actinobacteria isolates from marine environments contain polyketide synthetase (PKS) and nonribosomal polyketide synthetase (NRPS) pathways, the characteristics of secondary metabolites production [16].

The Tanjung Lumpur mangrove forest located on the east coast of Peninsular Malaysia is mostly unexplored; therefore, this location is anticipated to be able to provide a rich source of Actinobacteria, the prolific producers of antimicrobial secondary metabolites. To our knowledge, no studies have reported the diversity and antimicrobial activities of Actinobacteria from Tanjung Lumpur mangrove environment. Therefore, there is a high possibility to identify novel Actinobacteria and discover valuable antimicrobial secondary metabolites. The aim of this study was to isolate and identify the Actinobacteria and screen them to discover potential sources for antimicrobial secondary metabolites.

## 2. Materials and Methods

### 2.1. Environmental Sampling

Soil sediments were collected from Tanjung Lumpur mangrove forest located in the city of Kuantan, State of Pahang, in December of the year 2012. Four different mangrove sediments were collected at site MUSC-TLS1 (3°48′3.2′′N 103°20′11.0′′E) until MUSC-TLS4 (3°48′21.3′′N 103°20′3.3′′E). At each site, five-sediment core samples were collected at a depth of 0–30 cm within a 50 m^2^ area. The sediments from each site were bulked and homogenized to prepare the composite samples. Sediments were placed into sterile plastic bags using an aseptic metal trowel and kept in the dark for transport to the laboratory. The physicochemical parameters such as temperature and pH of the sampling area were determined using soil temperature profile sensor ST01 and ph meter. There was little fluctuation in temperature and pH of the sampling locations. The temperature of soil samples ranged between 23 and 25°C and slightly acidic pH were observed in all the samples (6.1–6.4).

### 2.2. Selective Isolation of Actinobacteria

Air-dried soil sediment (~7 days) was ground with mortar and pestle. Selective pretreatment of the samples was performed using a phenol solution (1.5%, 30 min at 30°C) [17] or wet heat in sterilized water (15 min at 50°C) [18]. The pretreated samples were diluted 1 : 10 v/v with sterile 25% Ringer's solution and serial dilution to 10^−4^. One hundred *μ*L of the 10^−1^, 10^−2^, 10^−3^, and 10^−4^ suspensions was spread in triplicate onto isolation media.

Dilutions of soil suspensions were spread onto 6 different types of isolation media: ISP 2 (yeast malt agar), ISP 7 (tyrosine agar) [19], starch casein agar (SCA) [20],* Streptomyces* agar (SA) [21], Actinomycetes isolation agar (AIA) [21], and nutrient agar [22]. All media were supplemented with cycloheximide (50 mg/L), nystatin (50 mg/L), and nalidixic acid (20 mg/L) [23] and incubated at 28°C for 1–4 weeks. Purified cultures were maintained on ISP medium 2 [19] slants at room temperature for short-term storage and as glycerol suspensions (20%, v/v) at −80°C for long-term storage.

### 2.3. Morphological, Physiological, and Biochemical Characterizations of 87 Isolates of Actinobacteria

The cultural, morphological, biochemical, and physiological characterizations of the Actinobacteria isolates were performed as described by Shirling and Gottlieb [19]. Light microscopy (80i, Nikon) and scanning electron microscopy (JEOL-JSM 6400) were used to observe the morphologies of selected isolates after incubation on ISP 2 medium at 28°C for 7 days. Using the light microscope, the formation of aerial and substrate mycelium and spore arrangement was observed. Cultural characteristics of isolates, which include growth, colony color on different isolation media, the presence of aerial and substrate mycelium, distinctive reverse colony color, and diffusible pigment, were determined using six different isolation media including ISP 2, ISP 7, SCA, SA, AIA, and nutrient agar with procedures as described by International Streptomyces Project (ISP). The production of melanoid pigments was examined using tyrosine agar (ISP 7). The ISCC-NBS colour charts were used to determine the names and designations of the colony colours [24].

Biochemical characterizations such as Gram staining and blood hemolysis were performed. Gram staining was performed by standard Gram reaction and was confirmed by using KOH lysis [25]. Hemolytic activity was performed in blood agar medium containing 5% (w/v) peptone, 3% (w/v) yeast extract, 5% (w/v) NaCl, and 5% (v/v) human blood [26]. Plates were examined for hemolysis after incubation at 32°C for 5 days. The presence of clear zone around colonies signifies the potential of isolates for surfactant production. Physiological characterization such as growth temperature was performed. The growth temperature was tested at 12–52°C at intervals of 4°C on ISP 2. The Biolog GenIII MicroPlates were used according to manufacturer's instructions to determine a total of 71 carbon-source utilization assays and 23 chemical sensitivity assays for selected isolates.

### 2.4. Preliminary Screening of Actinobacteria Isolates for Antimicrobial Activity

Eighty-seven isolates were preceded to preliminary screening for antimicrobial activity by using the cross streak method [27]. The isolates were cross streaked on ISP 2 medium and incubated at room temperature for 5–7 days. After observing a good growth of Actinobacterial cultures, overnight cultures of 12 different pathogens were used for the screening; namely,* Bacillus subtilis* ATCC 31098^T^,* Bacillus cereus* NBRC 13494^T^,* Enterococcus faecalis* NBRC 12965^T^, methicillin-resistant* Staphylococcus aureus* (MRSA) ATCC BAA-44^T^,* Staphylococcus epidermidis* ATCC 12228^T^,* Aeromonas hydrophila* ATCC 7966^T^,* Acinetobacter calcoaceticus* NBRC 13006^T^,* Klebsiella oxytoca* NBRC 12582^T^,* Klebsiella pneumonia* NBRC 14440^T^,* Pseudomonas aeruginosa* NRBC 12582^T^,* Salmonella typhi* ATCC 19430^T^, and* Yersinia pseudotuberculosis* NBRC 105692^T^ were streaked at the right angle of Actinobacterial cultures. Plates were incubated at 28°C for 48 hrs and the zone of inhibition was recorded. ISP 2 plates without Actinobacteria isolates but streaked with the same stock of pathogens were used as control.

### 2.5. Crude Extracts Preparation and Screening for Secondary Antimicrobial Metabolites

Eighty-seven Actinobacteria isolates were subjected to subsequent investigation by antimicrobial screening of their secondary metabolites. The method of Thakur et al. [31] was modified for screening bioactive antimicrobial metabolites. The same pathogens used during preliminary screening were used for this screening. These pathogens were cultured overnight at 37°C at 200 rpm in nutrient broth. The cultures were diluted with their respective media to 0.8–1.2 × 10^6^ CFU/mL, and 100 *μ*L aliquots of this inoculum were transferred to nutrient agar media.

The fermentation medium used was FM3 [32]; the medium was autoclaved at 121°C for 20 min. Each of the 87 purified isolates was transferred to a test tube (30 mm × 200 mm) containing 20 mL of the relevant fermentation medium and cultured at 200 rpm, at an angle of 45°, for 7–10 days at 28°C. The resulting fermentation media obtained from each of the isolates were separated from the mycelium by centrifugation at 10,000 rpm at 4°C for 15 min. The supernatants were filtered (Whatman number 1 filter paper) and tested for extracellular antimicrobial activity against different target pathogens using agar well diffusion method [33]. A sterile cork borer was used to puncture well in appropriate agar medium plates previously seeded with one of the pathogen strains. One hundred *μ*L from the fermentation supernatant of the isolates was added to each of the wells. The inoculated plates were kept at 4°C for at least 2 hrs to allow the diffusion of produced antimicrobial metabolites. The diameters of inhibition are determined after 24 hrs of incubation at 37°C and verified active substance extraction. Each experiment was repeated three times and average value of inhibitory zones was reported. Blank wells without the fermentation medium were taken as control.

### 2.6. Molecular Identification of Actinobacteria Isolates

#### 2.6.1. Genomic DNA Extraction and PCR Amplification of 16S rDNA

Genomic DNA extractions for 87 isolates of Actinobacteria were performed as described by Hong et al. [1]. The primer pair 27F-1492R [1, 28] was used for PCR amplification using the Kyratec PCR Supercycler (Kyratec, Australia). The PCR reaction mixture that consisted of 20–200 ng bacteria genomic DNA, 10.0 *μ*L of 2X Prime Taq Premix (Genet Bio, Korea), 10 pmoles primer 27F and primer 1492R, and sterile ultrapure water was added to final volume of 20 *μ*L. The cycling parameters were as described by Hong et al. [1].

#### 2.6.2. Phylogenetic Analysis of 16S rRNA Gene Sequences

The PCR products were purified using the GeneAll Expin Gel SV purification kit (GeneAll, South Korea); and then molecular cloning was performed using the InsTAclone PCR cloning kit (Thermo Scientific, USA) according to the manufacturer's protocols. The insertions were verified using colony-PCR and colonies with transformations were preceded to plasmid DNA extraction using the Eppendorf FastPlasmid Minikit. Purified plasmid DNA served as templates for PCR to confirm the insertion of the gene of interest. Plasmid DNAs were sequenced using an ABI PRISM 3100 DNA sequencer (Applied Biosystems, USA).

The cloned 16S rRNA gene sequences were aligned manually using sequences from the closest related genera retrieved from the GenBank/EMBL/DDBJ databases using CLUSTAL-X [34]. The alignment was manually verified and adjusted prior to the construction of a phylogenetic tree. The calculation for the level of sequence similarity was performed using the EzTaxon-e server (http://eztaxon-e.ezbiocloud.net/) [35]. The phylogenetic tree was inferred using the neighbor joining algorithms [36] via molecular evolutionary genetic analysis (MEGA version 5.2) [37]. The stabilities of the resultant tree topologies were evaluated using bootstrap analysis [38]. The pairwise distances between sequences were generated using Kimura's 2-parameter model [39].

#### 2.6.3. DNA-DNA Hybridization for Putative Novel Isolates

Biomass for chemotaxonomic studies was obtained after growing in tryptic soy broth (TSB) at 28°C for 7 days on a rotary shaker. The extraction of genomic DNA for DNA-DNA hybridization of putative novel isolates (MUSC 115^T^ and MUSC 135^T^) and their closely related type strains was carried out by the identification service of the DSMZ, Braunschweig, Germany. Genomic DNA extractions from the isolates were performed as described by Cashion et al. [40]. DNA-DNA hybridization was carried out as described by de Ley et al. [41] under consideration of the modifications described by Huss et al. [42] using a model Cary 100 Bio UV/VIS-spectrophotometer equipped with a Peltier-thermostatted 6 × 6 multicell changer and a temperature controller with* in situ* temperature probe (Varian).

#### 2.6.4. PCR Detection of PKS-I, PKS-II, and NRPS Sequences

Three sets of degenerate primers were used for the amplification of genes encoding polyketide synthases I and II (PKS-I and PKS-II) and nonribosomal peptide synthetases (NRPS) (Table 1). The PCR reaction mixture that consisted of 20–200 ng bacteria genomic DNA, 10.0 *μ*L of 2X Prime Taq Premix (Genet Bio, Korea), 10 pmoles of different primer sets (Table 1), and sterile ultrapure water was added to final volume of 20 *μ*L. The PCR was performed using the Kyratec PCR Supercycler (Kyratec, Australia) with the following cycling conditions: (i) 94°C for 5 min; (ii) 30 cycles of 94°C for 1 min, 57°C (for K1F-M6R and KS*α*-KS*β*) or 62°C (for A3F-A7R) for 1 min, and 72°C for 2 min; and (iii) 72°C for 5 min. The PCR amplification products were resolved using electrophoresis in 1.5% agarose gel (Promega, USA) and stained with ethidium bromide (0.5 *μ*g mL^−1^) and viewed using Molecular Imager Chemidoc XRS System (Biorad, USA).

## 3. Results and Discussion

### 3.1. Selective Isolation of Actinobacteria

Based on the distinct morphology of Actinobacteria isolates on the media plate, a total of 87 isolates of Actinobacteria were successfully isolated from four composite mangrove sediments collected from Tanjung Lumpur of the State of Pahang, Malaysia. Sediments samples were named as TLS1, TLS2, TLS3, and TLS4 that were contributing to 47, 12, 15, and 13 isolates, respectively, of the total number of isolates. Eighty-seven isolates were isolated from 6 types of isolation media supplemented with cycloheximide, nystatin, and nalidixic acid; these media are, namely, SCA (*n* = 35), ISP 2 (*n* = 18), ISP 7 (*n* = 18), AIA (*n* = 11), NA (*n* = 3), and SA (*n* = 2). These results indicated that starch casein agar (SCA) was the most suitable medium for isolating Actinobacteria in this study, and this result is in agreement with others [43].

### 3.2. Diversity of Actinobacteria Isolates

Eighty-seven Actinobacteria isolates were identified to the genera level based on the molecular and morphological characteristics. The 16S rRNA gene sequences of these isolates were compared with 16S rRNA sequences of type strains retrieved from DDBJ/EMBL/GenBank. Results showed that Actinobacteria isolated in this study exhibited high level of diversity, as the 87 isolates were distributed among 5 suborders: Streptomycineae (*n* = 53), Micrococcineae (*n* = 16), Corynebacterineae (*n* = 16), Micromonosporineae (*n* = 1), and Propionibacterineae (*n* = 1) within the phylum Actinobacteria. A total of 11 genera were identified and each genus was distinguished by its 16S rRNA gene sequences. Of the 87 isolates, 59.8% (*n* = 52) of the isolates were assigned as genus* Streptomyces*, 14.9% (*n* = 13) as the genus* Mycobacterium,* and the remaining isolates as* Leifsonia* (*n* = 6),* Microbacterium* (*n* = 4),* Sinomonas* (*n* = 4),* Nocardia* (*n* = 2),* Terrabacter* (*n* = 2),* Gordonia* (*n* = 1),* Micromonospora* (*n* = 1),* Nocardioides* (*n* = 1), and* Streptacidiphilus* (*n* = 1). In this study, it is evident that some genera not commonly found in the mangrove environment were discovered, such as* Streptacidiphilus*,* Sinomonas*,* Terrabacter,* and* Leifsonia*. So far, the different species of* Streptacidiphilus* were isolated from area such as* Pinus* soils [44], acidic rhizosphere soil [45], and rice field soil [46], while* Sinomonas* was mostly discovered in forest soils [47], polluted forest soil [48], and volcanic soil [49].

The isolation of Actinobacteria from eleven genera showed a wide distribution of Actinobacteria in mangrove environment, especially in soil and sediments [1]. The presence of predominant number of* Streptomyces* isolates (59.8%) in this study is in agreement with results reported by Hong et al. [1] which isolated substantial* Streptomyces* isolates from mangrove soils in China. The identification of substantial number of* Streptomyces* isolates was extremely important for the antimicrobial bioactivities screening in this study, as this genus is proven to be the prolific producers of novel antibiotics [50, 51], with approximately 75% of commercially useful antibiotics being derived from* Streptomyces* [5].

**Figure 1:**
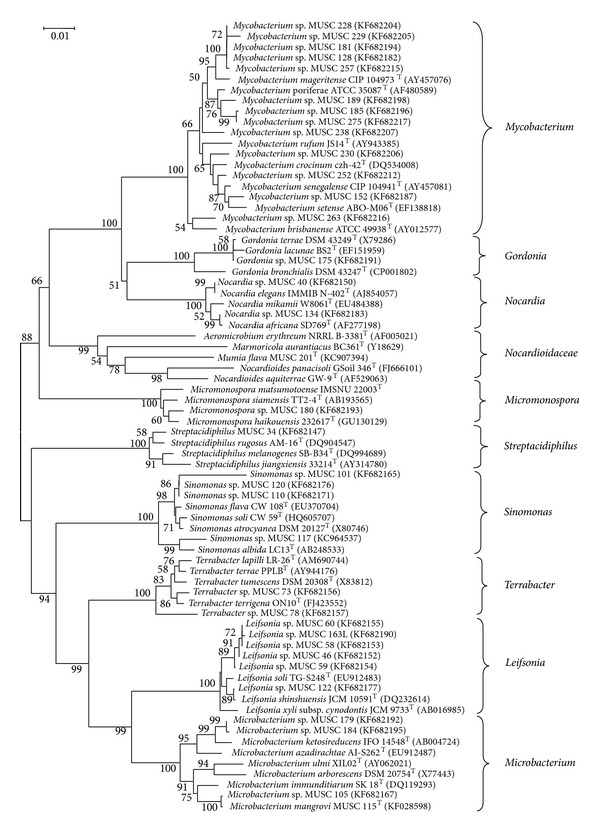
Phylogenetic tree based on 16S rRNA sequences using neighbour-joining method for 35 isolates of non-*Streptomyces* Actinobacteria and their closely related type strains. Bootstrap values (>40%) based on 1000 resampled datasets are shown at branch nodes. Bar, 1 substitution per 100 nucleotide positions.

**Figure 2:**
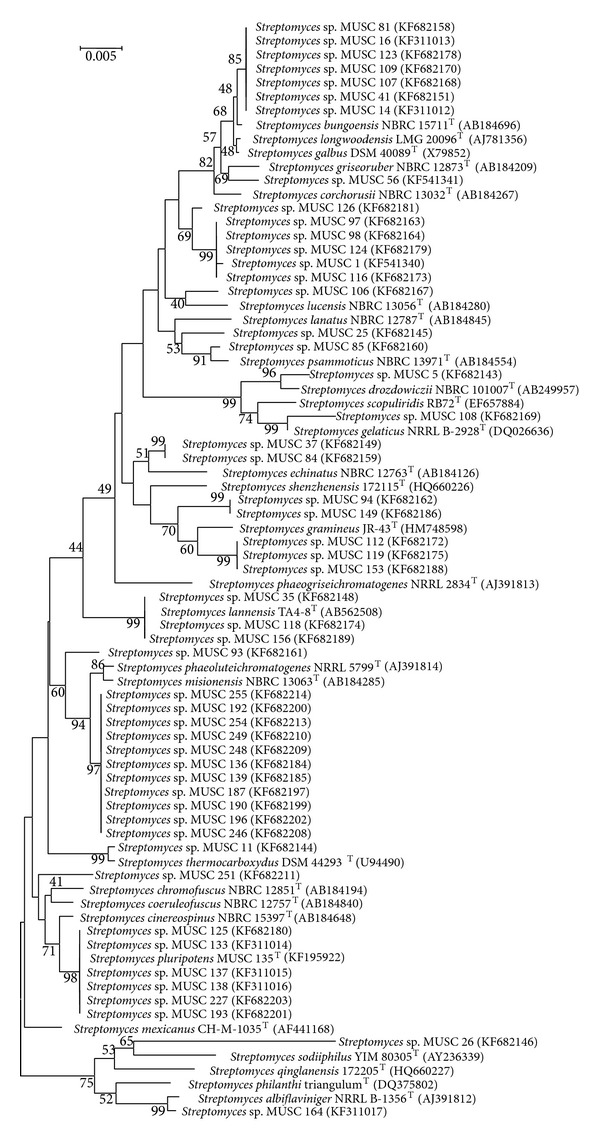
Phylogenetic tree based on 16S rRNA sequences using neighbour-joining method for 52 isolates of* Streptomyces* sp. and their closely related type strains. Bootstrap values (>40%) based on 1000 resampled datasets are shown at branch nodes. Bar, 5 substitutions per 1000 nucleotide positions.

The analysis of 16S rRNA sequences is used to determine higher taxonomic relationships of Actinobacteria [52, 53]. In this study, the 16S rRNA gene sequences were used to build a phylogenetic tree for 35 isolates of non-*Streptomyces* isolates together with their closely related type strains (Figure 1), whereas another phylogenetic tree was built for 52 isolates assigned to genus* Streptomyces* and their closely related type strains (Figure 2). The percentages of the 16S rRNA gene sequence similarities (95.1 to 100%) of these isolates to the closest type strains are shown in Table 2. The taxonomic studies showed that some isolates were highly potential to be assigned as novel genus or species based on the phylogenetic and pairwise comparison of 16S rRNA gene sequences of the novel isolates with the type strains. Four isolates from three genera (*Sinomonas*,* Microbacterium,* and* Streptomyces*) showed high possibilities of novel species discovery and one isolate (MUSC 201^T^) was identified as a novel genus in the family* Nocardioidaceae*.

Isolate MUSC 201^T^ was obtained from soil sample MUSC-TLS4 (3°48′21.3′′N 103°20′3.3′′E) pretreated with wet heat method [18]; the mixture was spread onto ISP 2 supplemented with cycloheximide and nystatin and incubated at 28°C for 7 days. The 16S rRNA gene sequences were determined for isolate MUSC 201^T^ (1486 bp) [GenBank: KC907394]. The comparison of isolate MUSC 201^T^ to the closely related phylogenetic neighbors indicated that it was closely related to the type strains of different genera within the family* Nocardioidaceae*:* Nocardioides* (95.1% to 91.9% similarity),* Aeromicrobium* (94.6 to 92.7% similarity),* Marmoricola* (93.1% to 92.5% similarity), and* Kribbella* (92.4% to 91.5% similarity). Isolate MUSC 201^T^ formed a distinct monophyletic clade within the family* Nocardioidaceae* (Figure 1), most closely related to type strain* Nocardioides panacisoli* GSoil 346^T^, and Actinobacteria were isolated from soil of ginseng field [54] at high bootstrap value of 78%. Isolate MUSC 201^T^ had low 16S rRNA gene sequence similarities with members of the closest related genus* Nocardioides* (94.1–95.1%) and was separated from them by a long evolutionary distance in the phylogenetic tree (Figure 1). On the basis of phylogenetic, genotypic, and chemotaxonomic profiles, isolate MUSC 201^T^ is truly different from any existing genera in the family* Nocardioidaceae*; hence, isolate MUSC 201^T^ represented a novel species in a new genus of the family* Nocardioidaceae*, for which the name “*Mumia flava* gen. nov., sp. nov.” is proposed and published [55].

The* Microbacterium* sp. isolates (MUSC 179, MUSC 184, MUSC 105, and MUSC 115^T^) formed distinct phylogenetic clade in the neighbour-joining tree but were separated from other members of the genus* Microbacterium* (Figure 1). Isolates MUSC 179 and MUSC 184 were closely related to* Microbacterium ketosireducens* IFO 14548^T^ (97.7% similarity) and* Microbacterium azadirachtae* AI-S262^T^ (97.7% similarity) (Table 2). Another 2 isolates (MUSC 105 and MUSC 115) were assigned to* Microbacterium immunditiarum* SK 18^T^, with 98.0 and 98.1% similarity (Table 2). Isolate MUSC 115^T^ was selected for further analysis to determine its potential as novel species.

Isolate MUSC 115^T^ was obtained from soil sample MUSC-TLS1 (3°48′3.2′′N 103°20′11.0′′E) pretreated with wet heat method [18]; the mixture was spread onto starch casein agar. The 16S rRNA gene sequence was established for strain MUSC 115^T^ (1484 bp) [GenBank: KF028598]. Phylogenetic analysis of isolate MUSC 115^T^ indicated that it formed a subclade with* Microbacterium immunditiarum* SK 18^T^ at high bootstrap supported value of 75% (Figure 1). Pairwise comparison of the 16S rRNA gene sequences from isolate MUSC 115^T^ showed similarities to* Microbacterium immunditiarum* SK 18^T^,* Microbacterium ulmi *XIL02^T^, and* Microbacterium arborescens* NBRC 3750^T^ at 98.1, 97.8, and 97.5%, respectively. The DNA-DNA relatedness values between strain MUSC 115^T^ and* Microbacterium immunditiarum* SK 18^T^ (23.6 ± 0.5%),* Microbacterium ulmi *XIL02^T^ (26.2 ± 2.7%), and* Microbacterium arborescens* DSM 20754^T^ (16.3 ± 1.1%) were significantly lower than 70%, the threshold value for the delineation of genomic species [56]. The DDH results suggested that strain MUSC 115^T^ does not belong to any of these species. The comparison of phenotypic characterization between isolate MUSC 115^T^ with its closely related type strains showed substantial differences between them [57]. Hence, isolate MUSC 115^T^ represented a novel species within the genus* Microbacterium* with lineages distinct from other members of the genus* Microbacterium* (Figure 1), proposed as “*Microbacterium mangrovi *sp.* nov*.” [57].

The* Sinomonas* sp. isolates (MUSC 101, MUSC 110, MUSC 117, and MUSC 120) formed a distinct phylogenetic clade that was distinct from other members of the phylum Actinobacteria with high bootstrap value (100%) (Figure 1). Two isolates (MUSC 101 and MUSC 120) were assigned to* Sinomonas soli*, with 96.8 and 99.1% similarity (Table 2). Isolate MUSC 101 was separated from the rest of the isolates by a long evolutionary distance in the phylogenetic tree; this association is supported by bootstrap value of 86% (Figure 1). The 16S rRNA gene sequence similarity value was lower than 97%, which is considered to be the cutoff value for species identity [58]. Therefore, isolate MUSC 101 could serve as a highly potential candidate as a novel species of the genus* Sinomonas.* Phylogenetic analysis of isolates* Sinomonas* sp. MUSC 117 indicated that it formed a distinct clade with type strains from genus* Sinomonas* at high bootstrap value of 100% (Figure 1). Isolate* Sinomonas* sp. MUSC 117 formed subclade with* Sinomonas albida*, an association supported by robust bootstrap value of 99% (Figure 1). The pairwise comparison of 16S rRNA gene sequence analysis showed that isolate MUSC 117 is assigned to type strains* Sinomonas atrocyanea*,* Sinomonas albida,* and* Sinomonas soli* with 98, 97.9, and 97.8% similarity, respectively. These 16S rRNA gene similarity values are less than the similarity values between closely related* Sinomonas* species, such as* S. atrocyanea* and its closest related species,* S. soli* (99.5% similarity). Therefore, isolate MUSC 117 serves as a good candidate for novel species in the genus* Sinomonas*.

### 3.3. Diversity and Phylogeny of Bioactive Actinobacteria Isolates

Of 87 isolates, 9 isolates which belonged to genus* Streptomyces* were producing bioactive metabolites (Table 2). The relationships between taxonomic and metabolic diversity are being highlighted for these bioactive isolates. As more novel Actinobacteria isolated from various environments are able to offer precious sources of new bioactive metabolites and compounds [59–61], the analysis of the 16S rRNA gene sequences of the bioactive isolates showed that isolates* Streptomyces* sp. MUSC 14 and* Streptomyces* sp. MUSC 16 shared the highest similarities to* Streptomyces bungoensis* NBRC 15711^T^ with 99.5 to 99.4% identities (Table 2). They formed distinct subclade with* Streptomyces bungoensis* NBRC 15711^T^ at 48% bootstrap value (Figure 2). Isolate MUSC 164 showed 98.4% identities to* Streptomyces albiflaviniger* NRRL B-1356^T^ (Table 2) and they formed a monophyletic clade at 99% bootstrap value (Figure 2).

Isolates* Streptomyces* sp. MUSC 133, MUSC 135^T^, MUSC 137, and MUSC 138 exhibited the highest similarities to sequences of type strain* Streptomyces cinereospinus* with 99.2 to 99.3% identities (Table 2). The phylogenetic analysis showed that isolates MUSC 133, MUSC 135^T^, MUSC 137, and MUSC 138 formed a distinct clade with type strains* Streptomyces cinereospinus* NBRC 15397^T^ at bootstrap value of 71% (Figure 2). Furthermore, these* Streptomyces* isolates (MUSC 133, MUSC 135^T^, MUSC 137, and MUSC 138) formed a distinct monophyletic subclade supported by robust bootstrap values of 98% (Figure 2). The 16S rRNA gene sequence similarities between isolate MUSC 135^T^ and isolates MUSC 133, MUSC 137, and MUSC 138 were 100, 100, and 99.9%, respectively. Therefore, isolates MUSC 133, MUSC 135^T^, MUSC 137, and MUSC 138 should be considered as the same species. Isolate MUSC 135^T^ was chosen as the representative for subsequent characterization as this isolate could produce diverse and potent antimicrobial secondary metabolites. Isolate MUSC 135^T^ showed similarities to type strains* Streptomyces cinereospinus* NBRC 15397^T^,* Streptomyces mexicanus* CH-M-1035^T^, and* Streptomyces coeruleofuscus* NBRC 12757^T^ with 99.18, 99.17, and 98.97% similarity, respectively (Table 2). These similarities values are lower as compared to the similarity value of type strain* Streptomyces cinereospinus* NBRC 15397^T^ to its closest related species* Streptomyces coeruleofuscus* NBRC 12757^T^ (99.4% similarity). Furthermore, the DNA-DNA relatedness values between strain MUSC 135^T^ and* Streptomyces cinereospinus* NBRC 15397^T^ (26.3 ± 2.1%),* coeruleofuscus* NBRC 12757^T^ (49.5 ± 2.9%), and* Streptomyces mexicanus* NBRC 100915^T^ (49.6 ± 2.5%) were notably below 70%, the threshold value for the delineation of genomic species [56]. Polyphasic characterizations results supported the observation that isolate* Streptomyces* sp. MUSC 135^T^ represented a novel species, proposed as “*Streptomyces pluripotens* sp. nov.,” [62] that exhibited merits in producing antimicrobial secondary metabolites.

### 3.4. Antibacterial Potential of Isolates

The 87 isolates were preceded to preliminary screening for antimicrobial activity against 12 pathogens by using the cross streak method [31]. Of 87 isolates, 48 isolates (55.2%) exhibited inhibitory against at least 1 pathogen used in this study. Of 48 isolates, 20.8% (*n* = 10) exhibited inhibitory activity against Gram negative bacteria and 2.1% (*n* = 1) inhibited Gram positive bacteria. The remaining 77.1% (*n* = 37) showed excellent inhibition towards both Gram negative and Gram positive bacteria.

### 3.5. Antimicrobial Activity of Culture Extracts from Mangrove Actinobacteria Isolates

The 87 Actinobacteria isolates were subjected to subsequent investigation by antimicrobial screening of their secondary metabolites. The secondary metabolites of these isolates were tested for antimicrobial activities against the same group of 12 pathogenic bacteria used during preliminary screening. Of 87 isolates, 9 isolates (10.3%) which belonged to the genus* Streptomyces* were exhibiting activity against at least one of the pathogenic bacteria tested. Of the 9 isolates, 55.6% (*n* = 5) were active against more than 1 pathogen. Two isolates (MUSC 14 and MUSC 16) were inhibiting 2 pathogens. Three isolates (MUSC 56, MUSC 135, and MUSC 164) exhibited broad spectrum of antibacterial activity, with MUSC 135 showing the strongest inhibition effects against MRSA (inhibition zone of 12 mm),* Bacillus cereus* (4 mm),* Acinetobacter calcoaceticus* (4 mm), and* Salmonella typhi* (4 mm) in this study (Table 2). The strong inhibitory activities of these Actinobacteria isolates towards an array of pathogens are a good indicator that these isolates could be the potential candidates for production of highly valuable bioactive secondary metabolites. Some of the bioactive metabolites are currently being chemically analyzed to identify the novelty of the active compounds.

The identification of* Streptomyces* as the most bioactive genus in this study is in line with other researchers [1] as* Streptomyces* has the ability to catabolize a wide range of compounds and produce secondary metabolites with diverse biological activities and chemical structure [4].* Streptomyces* is the largest genus of the Actinobacteria and over two-thirds of all natural antibiotics are derived from this group of bacteria [5].* Streptomyces* has a huge biosynthetic potential that remains unchallenged among other microbial groups. This is proven as some* Streptomyces* species, whose biosynthetic repertoire was considered to be only three to five secondary metabolites, in fact possess more than 20 genomic regions encoding known or predicted biosynthetic pathways [63, 64].

Total of 10% (9/87) of Actinobacteria isolates exhibited antimicrobial properties in this study. These results indicated that Actinobacteria are still capable of producing highly bioactive metabolites and continued to provide high quality biological material for drug discovery. Nevertheless, to increase the chances of discovering bioactive isolates in future study, new targets could be used to detect more activities such as antimicrobial and cytotoxic activities [65], as the discovery of novel microbial natural products is encouraged not only by the quality of biological material but also by the innovation and sensitivity of the screening models used [1].

### 3.6. Detection of PKS and NRPS Genes in Actinobacteria Isolates

Many Actinobacterial isolates from marine environments contain polyketide synthetase (PKS) and nonribosomal polyketide synthetase (NRPS) pathways, the characteristics of secondary metabolite production [16]. In this study, eighty-seven isolates were screened for the presence of PKS-I, PKS-II, and NRPS gene sequences by specific amplification of chromosomal DNA with primer sets K1F-M6R, KS*α*-KS*β*, and A3F-A7R (Table 1). The amplification products were examined by 1% agarose gel electrophoresis, and bands of 1200 to 1400 bp, 600 bp, and 700 to 800 bp were identified as products of PKS-I, PKS-II, and NRPS genes, respectively (Table 1). Of 87 isolates tested for the presence of PKS and NRPS genes, 52 isolates (59.7%) exhibited at least one type of biosynthetic sequences. PKS-II genes were the most frequent as it is detected in 42.5% (*n* = 37) of the isolates. PKS-I genes were detected in 19.5% of the isolates (*n* = 17), and NRPS genes were exhibited by only 5 isolates (5.7%). The only isolate that contained all of the biosynthetic genes (PKS-I, PKS-II, and NRPS) used is MUSC 134, a* Nocardia* species that is closely related to* Nocardia Africana* at 99.8% similarity. Isolates MUSC 119, MUSC 192, and MUSC 249 contained both of the PKS-I and PKS-II genes, whereas isolates MUSC 81 and MUSC 152 showed positive amplification of both PKS-I and NRPS genes products.

The percentage of bioactive isolates (10.3%) detected in this study is relatively low as compared to the percentage of isolates with at least one type of biosynthetic sequences (59.7%). This showed that the fermentation media (FM3) used may not be able to provide the nutrients and conditions needed to stimulate secondary metabolite production in many of the mangrove Actinobacteria isolates in this study. Therefore, the PCR detection of polyketide synthase types I and II and the nonribosomal peptide synthetase genes is vital to discover the potential of these isolates to produce valuable secondary metabolites. Some of the secondary metabolite biosynthetic gene clusters appear to be nonactive or silent under standard culture conditions and they may require some specific triggers to be activated [66]; these data exhibited that the nonbioactive isolates hold the genetic capability to produce useful secondary metabolites if they were cultivated under the appropriate conditions. As rare taxa such as the* Nocardioides* (isolate MUSC 201^T^) showed biosynthetic potential by carrying the PKS-II genes, shedding light on the possibility to serve as a source of natural products, but even this genus has not been shown to produce any natural products yet.

The absence of amplification products from some of the isolates may reveal the lack of PKS-I, PKS-II, and NRPS genes. However, the negative results could be caused by the types of degenerate primers used, which were not suitable for amplifying these genes [67], especially for the low detection rate of NRPS genes in this study. Moreover, it is reported that not all of the NRPS genes are involved in the biosynthesis of bioactive secondary metabolites, as these genes could be responsible in functionality of the bacteria such as quorum sensing [68] or iron metabolism [30]. The high rate of detection of PKS-I and PKS-II genes in the isolates tested was mostly* Streptomyces*, providing strong evidence for the high potential of* Streptomyces* to produce high number of biologically active metabolites. Therefore, the molecular screening of Actinobacteria isolates for genes encoding biosynthesis of bioactive compounds is still an effective and valuable approach for preselecting isolates for useful secondary metabolites production [29, 67, 69–73].

## 4. Conclusions

In conclusion, this study performed a comprehensive investigation into the diversity, phylogeny, and biosynthetic and antimicrobial potential of Actinobacteria isolated from tropical mangrove sediments in east coast of Peninsular Malaysia. A substantial diversity of Actinobacteria isolates was isolated from mangrove habitats, and it is apparent that isolates from genera such as* Sinomonas*,* Microbacterium*, and* Streptomyces* could merit novel species status. Many of the Actinobacteria isolates are producing bioactive secondary metabolites or possess detectable biosynthetic genes, indicating that mangrove habitats are a valuable source of discovery for novel Actinobacteria with promising potential to produce highly bioactive antimicrobial metabolites that could have important value in drug discovery programs.

## Figures and Tables

**Table 1:** PCR primers used in this study.

Primer name	Sequence (5′-3′)	Target gene	Product size (bp)	Reference
27F	GTTTGATCCTGGCTCAG	16S rRNA	1400–1500	[1, 28]
1492R	TACGGCTACCTTGTTACGACTT			
K1F	TSAAGTCSAACATCGGBCA	PKS-I	1200–1400	[29]
M6R	CGCAGGTTSCSGTACCAGTA			
KS*α*	TSGCSTGCTTGGAYGCSATC	PKS-II	600	[30]
KS*β*	TGGAANCCGCCGAABCCTCT			
A3F	GCSTACSYSATSTACACSTCSGG	NRPS	700–800	[29]
A7R	SASGTCVCCSGTSCGGTAS			

**Table 2:** Identification and antimicrobial activities and presence of PKS and NRPS genes in Actinobacteria isolates from Tanjung Lumpur, Malaysia.

Isolate number (Genbank accession number)	Closest relative	Sequence identity (%)	Activity^a^ (mm) against pathogens	Presence of gene		
				PKS-I	PKS-II	NRPS
MUSC 1 (KF541340)	*Streptomyces corchorusii *	98.8	BS (9)	+	−	−
MUSC 5 (KF682143)	*Streptomyces drozdowiczii *	99.4	−	−	−	−
MUSC 11 (KF682144)	*Streptomyces thermocarboxydus *	99.9	−	−	+	−
MUSC 14 (KF311012)	*Streptomyces bungoensis *	99.4	MRSA (2), SE (2)	+	−	−
MUSC 16 (KF311013)	*Streptomyces bungoensis *	99.5	MRSA (3), SE (3)	+	−	−
MUSC 25 (KF682145)	*Streptomyces philanthi *	96.3	−	−	−	−
MUSC 26 (KF682146)	*Streptomyces qinglanensis *	96.2	−	−	−	−
MUSC 34 (KF682147)	*Streptacidiphilus rugosus *	99.4	−	−	−	−
MUSC 35 (KF682148)	*Streptomyces lannensis *	100.0	−	−	+	−
MUSC 37 (KF682149)	*Streptomyces phaeogriseichromatogenes *	97.9	−	−	−	−
MUSC 40 (KF682150)	*Nocardia elegans *	99.9	−	−	+	−
MUSC 41 (KF682151)	*Streptomyces bungoensis *	99.6	−	+	−	−
MUSC 46 (KF682152)	*Leifsonia shinshuensis *	99.4	−	−	−	−
MUSC 56 (KF541341)	*Streptomyces antibioticus *	100.0	BS (4.5), BC (4), EF (3)	−	+	−
MUSC 58 (KF682153)	*Leifsonia shinshuensis *	99.3	−	−	−	−
MUSC 59 (KF682154)	*Leifsonia shinshuensis *	99.3	−	−	−	−
MUSC 60 (KF682155)	*Leifsonia shinshuensis *	99.4	−	−	−	−
MUSC 73 (KF682156)	*Terrabacter tumescens *	99.0	−	−	−	−
MUSC 78 (KF682157)	*Terrabacter lapilli *	98.3	−	−	−	−
MUSC 81 (KF682158)	*Streptomyces bungoensis *	97.8	−	+	−	+
MUSC 84 (KF682159)	*Streptomyces echinatus *	98.7	−	−	−	−
MUSC 85 (KF682160)	*Streptomyces psammoticus *	99.5	−	−	+	−
MUSC 93 (KF682161)	*Streptomyces phaeogriseichromatogenes *	97.0	−	−	+	−
MUSC 94 (KF682162)	*Streptomyces gramineus *	98.6	−	−	−	−
MUSC 97 (KF682163)	*Streptomyces phaeogriseichromatogenes *	97.7	−	+	−	−
MUSC 98 (KF682164)	*Streptomyces corchorusii *	98.9	−	−	−	−
MUSC 101 (KF682165)	*Sinomonas soli *	96.8	−	−	−	−
MUSC 105 (KF682166)	*Microbacterium immunditiarum *	98.0	−	−	−	−
MUSC 106 (KF682167)	*Streptomyces lucensis *	99.2	−	−	+	−
MUSC 107 (KF682168)	*Streptomyces bungoensis *	98.8	−	+	−	−
MUSC 108 (KF682169)	*Streptomyces scopuliridis *	97.1	−	−	+	−
MUSC 109 (KF682170)	*Streptomyces bungoensis *	99.6	−	+	−	−
MUSC 110 (KF682171)	*Sinomonas atrocyanea *	99.4	−	−	−	−
MUSC 112 (KF682172)	*Streptomyces gramineus *	98.4	−	−	+	−
MUSC 115 (KF028598)	*Microbacterium immunditiarum *	98.1	−	−	−	−
MUSC 116 (KF682173)	*Streptomyces phaeogriseichromatogenes *	97.3	−	−	−	−
MUSC 117 (KC964537)	*Sinomonas atrocyanea *	98.0	−	−	−	−
MUSC 118 (KF682174)	*Streptomyces lannensis *	97.4	−	−	−	−
MUSC 119 (KF682175)	*Streptomyces gramineus *	97.8	−	+	+	−
MUSC 120 (KF682176)	*Sinomonas soli *	99.1	−	−	−	−
MUSC 122 (KF682177)	*Leifsonia soli *	99.5	−	−	−	−
MUSC 123 (KF682178)	*Streptomyces bungoensis *	97.1	−	+	−	−
MUSC 124 (KF682179)	*Streptomyces phaeogriseichromatogenes *	97.9	−	−	−	−
MUSC 125 (KF682180)	*Streptomyces cinereospinus *	99.3	−	−	+	−
MUSC 126 (KF682181)	*Streptomyces phaeogriseichromatogenes *	98.3	−	+	−	−
MUSC 128 (KF682182)	*Mycobacterium rufum *	96.7	−	−	−	−
MUSC 133 (KF311014)	*Streptomyces cinereospinus *	99.3	MRSA (6)	−	+	−
MUSC 134 (KF682183)	*Nocardia africana *	99.8	−	+	+	+
MUSC 135 (KF195922)	*Streptomyces cinereospinus *	99.2	BC (4), MRSA (12), AH (4), ST (4)	−	+	−
MUSC 136 (KF682184)	*Streptomyces phaeoluteichromatogenes *	98.5	−	−	+	−
MUSC 137 (KF311015)	*Streptomyces cinereospinus *	99.2	MRSA (4)	−	+	−
MUSC 138 (KF311016)	*Streptomyces cinereospinus *	99.2	MRSA (11)	−	+	−
MUSC 139 (KF682185)	*Streptomyces phaeogriseichromatogenes *	97.5	−	−	+	−
MUSC 149 (KF682186)	*Streptomyces gramineus *	97.9	−	−	+	−
MUSC 152 (KF682187)	*Mycobacterium setense *	97.9	−	+	−	+
MUSC 153 (KF682188)	*Streptomyces gramineus *	98.1	−	−	+	−
MUSC 156 (KF682189)	*Streptomyces lannensis *	97.3	−	−	+	−
MUSC 163L (KF682190)	*Leifsonia shinshuensis *	99.2	−	−	−	−
MUSC 164 (KF311017)	*Streptomyces albiflaviniger *	98.4	BC (2), EF (1), MRSA (4)	+	−	−
MUSC 175 (KF682191)	*Gordonia terrae *	99.9	−	−	−	−
MUSC 179 (KF682192)	*Microbacterium ketosireducens *	97.7	−	−	−	−
MUSC 180 (KF682193)	*Micromonospora siamensis *	97.9	−	−	+	−
MUSC 181 (KF682194)	*Mycobacterium rufum *	96.6	−	−	−	−
MUSC 184 (KF682195)	*Microbacterium azadirachtae *	97.7	−	−	−	−
MUSC 185 (KF682196)	*Mycobacterium crocinum *	97.9	−	−	+	−
MUSC 187 (KF682197)	*Streptomyces phaeogriseichromatogenes *	97.9	−	−	+	−
MUSC 189 (KF682198)	*Mycobacterium poriferae *	98.7	−	−	−	−
MUSC 190 (KF682199)	*Streptomyces phaeogriseichromatogenes *	97.9	−	−	−	−
MUSC 192 (KF682200)	*Streptomyces phaeoluteichromatogenes *	98.7	−	+	+	−
MUSC 193 (KF682201)	*Streptomyces mexicanus *	98.0	−	−	+	−
MUSC 196 (KF682202)	*Streptomyces phaeogriseichromatogenes *	97.8	−	−	+	−
MUSC 201 (KC907394)	*Nocardioides panacisoli *	95.1	−	−	+	−
MUSC 227 (KF682203)	*Streptomyces cinereospinus *	97.0	−	−	+	−
MUSC 228 (KF682204)	*Mycobacterium mageritense *	98.8	−	−	−	−
MUSC 229 (KF682205)	*Mycobacterium mageritense *	97.5	−	−	+	−
MUSC 230 (KF682206)	*Mycobacterium crocinum *	98.3	−	−	+	−
MUSC 238 (KF682207)	*Mycobacterium crocinum *	97.6	−	−	−	+
MUSC 246 (KF682208)	*Streptomyces phaeogriseichromatogenes *	97.5	−	−	+	−
MUSC 248 (KF682209)	*Streptomyces phaeogriseichromatogenes *	97.9	−	−	+	−
MUSC 249 (KF682210)	*Streptomyces misionensis *	98.3	−	+	+	−
MUSC 251 (KF682211)	*Streptomyces coeruleofuscus *	98.7	−	−	+	−
MUSC 252 (KF682212)	*Mycobacterium senegalense *	99.4	−	−	−	−
MUSC 254 (KF682213)	*Streptomyces phaeogriseichromatogenes *	97.5	−	+	−	−
MUSC 255 (KF682214)	*Streptomyces phaeogriseichromatogenes *	97.6	−	−	+	−
MUSC 257 (KF682215)	*Mycobacterium mageritense *	98.7	−	−	−	+
MUSC 263 (KF682216)	*Mycobacterium brisbanense *	98.2	−	−	−	−
MUSC 275 (KF682217)	*Mycobacterium poriferae *	99.0	−	−	−	−

^a^Activity estimated by measuring the diameter of the clear zone of the growth inhibition. −: no activity.

BS: *Bacillus subtilis*; BC: *Bacillus cereus*; EF: *Enterococcus faecalis*; MRSA: methicillin-resistant *Staphylococcus aureus*; SE: *Staphylococcus epidermidis*; AH: *Aeromonas hydrophila*; ST: *Salmonella typhi*.
